# Recent advances in the detection technologies for balanced chromosomal rearrangements

**DOI:** 10.3389/fgene.2026.1763846

**Published:** 2026-02-13

**Authors:** Meng Gao, Jun Ren, Shanling Liu

**Affiliations:** 1 Department of Medical Genetics, Center for Prenatal Diagnosis, West China Second University Hospital, Sichuan University, Chengdu, China; 2 Key Laboratory of Birth Defects and Related Diseases of Women and Children (Sichuan University), Ministry of Education, Chengdu, China

**Keywords:** balanced chromosomal rearrangements, clinical laboratory techniques, genome sequencing, molecular genetic testing, optical genome mapping

## Abstract

Balanced chromosomal rearrangements (BCRs) refer to a type of chromosomal structural variations without chromosomal gains or losses. BCR carriers may experience fertility issues, including a higher risk of infertility, recurrent miscarriages, or having offspring with chromosomal abnormalities. Since there are no apparent gains or losses of genetic materials in BCR carriers, their detection has long been a focal and challenging issue in the field of chromosomal structural variation analysis. Karyotyping cannot detect submicroscopic rearrangements because of restricted resolution and the application of fluorescence *in situ* hybridization (FISH) is limited by the necessity of a known loci. Chromosomal microarray analysis and standard short-read sequencing, widely used in clinical practice, cannot detect BCRs. In summary, the clinical detection techniques are unable to accurately identify the breakpoints of BCRs. The improved short-read sequencing such as mate-pair sequencing has been found to detect balanced rearrangements. Emerging advanced technologies such as long-read sequencing, and optical genome mapping, have already shown their potential in detecting BCRs. This review primarily elucidates the principles, applicability, advantages, and limitations of the detection techniques for BCRs, aiming to assist in the early identification and appropriate advice of patients with BCRs in genetic counseling.

## 1 Introduction

BCRs are a type of chromosomal structural rearrangement in which chromosomal segments break off and rejoin without large chromosomal gains or losses, including balanced translocations, inversions, and insertions. Complex BCRs involve combinations of such events between two or more chromosomes (Shen et al., 2024). Balanced translocations are the most common in clinical BCR carriers, with a prevalence of 1/500 to 1/625 in the general population, and up to 1/20 in patients with repeated *in vitro* fertilization (IVF) failure or recurrent miscarriage (Dong et al., 2019). Robertsonian translocations occur in about 1/1,000 newborns and approximately 1/800 in the general population, and in 0.65%–2.17% of patients with recurrent miscarriage (Poot and Hochstenbach, 2021; Zhang et al., 2021; Zhu et al., 2022). Chromosomal inversions are reported in 0.96%–1.10% of couples with recurrent miscarriage or undergoing IVF (Zhang et al., 2019).

BCR carriers are generally phenotypically normal, but have an increased risk of recurrent miscarriage, infertility, or affected offspring due to unbalanced gametes from aberrant meiotic segregation (Xia et al., 2023). A minority may develop neurodevelopmental or other neuropsychiatric disorders, possibly related to disruptions of gene regions or topologically associated domains (Zhang et al., 2023). Moreover, BCRs are not always truly balanced at the molecular level: cryptic copy-number variations near breakpoints can cause gene disruption or fusion, positional effects, or imprinting alterations, leading to abnormal phenotypes (Satkin et al., 2020).

**FIGURE 1:**
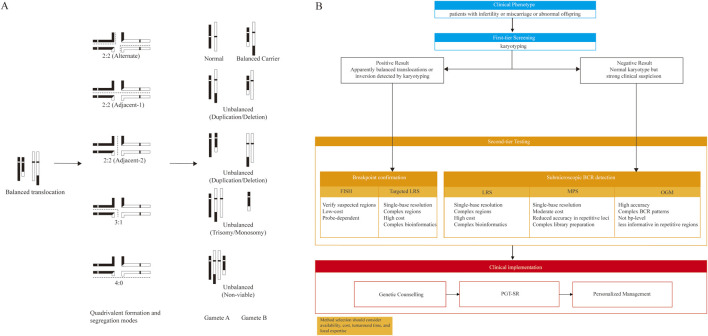
Meiotic segregation outcomes and a clinical workflow for BCR detection **(A)** In balanced translocation carriers, a quadrivalent forms at meiosis I and can segregate as 2:2 (alternate produces normal/balanced gametes; adjacent-1/adjacent-2 typically produce duplication/deletion), 3:1 (unbalanced), or 4:0 (generally non-viable). Representative gamete outcomes are shown **(B)** Proposed workflow: karyotyping as first-tier screening for infertility, miscarriage, or abnormal offspring; FISH/targeted LRS for breakpoint confirmation in karyotype-positive cases; MPS, OGM, or LRS for detecting cryptic BCRs in karyotype-negative cases with persistent suspicion, followed by genetic counselling, PGT-SR, and personalized management. BCR, balanced chromosomal rearrangement; FISH, fluorescence *in situ* hybridization; LRS, long-read sequencing; MPS, mate-pair sequencing; OGM, optical genome mapping; PGT-SR, preimplantation genetic testing for structural rearrangements.

In balanced translocation carriers, gamete formation depends on meiotic segregation patterns (Figure 1A). During pachytene, the four chromosomes form a quadrivalent, followed by three segregation modes: 2:2, 3:1, and 4:0. In 2:2 segregation, two chromosomes segregate to each daughter cell, producing alternate, adjacent-1, and adjacent-2 subtypes: alternate yields normal or balanced gametes, while adjacent-1 and adjacent-2 generate unbalanced gametes with duplication/deletion. In 3:1 segregation, three chromosomes segregate to one cell and one to the other, while 4:0 results in all four chromosomes moving to one cell, leaving the other empty (Scriven et al., 1998). In theory, heterozygous carriers can generate 16 unique zygotes from a balanced translocation, and up to 36 chromosomal combinations when mating with a normal partner. Most unbalanced gametes are nonviable, except for the two balanced gametes produced through 2:2 alternate segregation, with only specific trisomic gametes potentially surviving (Amor and Gardner, 2025). Therefore, accurate identification of BCR breakpoints is essential for elucidating the genetic implications.

Advances in molecular genetics have expanded the toolbox for BCR detection. Karyotyping remains the clinical gold standard for structural variations (SVs), but its resolution of 5–10 Mb limits detection of cryptic BCRs below this range. Fluorescence *in situ* hybridization (FISH) is targeted rather than genome-wide. Although chromosome microarray (CMA) improves detection of unbalanced aberrations, BCRs remain undetectable by microarray-based technologies (Levy and Wapner, 2018). Moreover, standard short-read paired-end sequencing is constrained by 150–300 bp fragment lengths, and many rearrangement breakpoints cannot be detected when breakpoint-spanning information exceeds the read or insert size. Mate-pair sequencing (MPS), as an optimized short-read strategy with larger insert sizes, partially alleviates these limitations for SVs discovery (Qian et al., 2025).

In recent years, long-read sequencing (LRS) and optical genome mapping (OGM) have emerged as promising approaches for detecting BCRs. In this review, we compare available technologies for BCR detection and summarize their clinical applications, emphasizing method-specific strengths and limitations.

## 2 Current methods for detecting balanced chromosome rearrangements

### 2.1 Cytogenetic methods

Karyotyping uses chromosome banding to visualize metaphase chromosomes and evaluate morphology (band pattern, length, and arm ratio) for detecting chromosomal abnormalities. Banding methods include G-, Q-, R-, C-, and N-banding, which are selected based on clinical indications. Among these, G-banding is the most widely used in clinical cytogenetics because it provides stable preparations with good banding resolution and can be analyzed with standard bright-field microscopy, enabling direct identification of BCRs (Karger, 2024).

G-banding is cost-effective and can detect apparent rearrangements, but its practical resolution is approximately 5–10 Mb (Levy and Wapner, 2018). Banding resolution is reported as bands per haploid set (e.g., 300–850) (Arsham et al., 2017). Higher-band karyograms can reveal subtler changes, with 550–700 bands capturing most abnormalities and 700–850 bands generally suitable for SVs > 3–5 Mb. High-resolution preparations can yield elongated or overlapping chromosomes, complicating interpretation. Analysis is labor-intensive, and turnaround is typically 7–14 days because cell culture is required (Saldarriaga et al., 2015). Interpretation depends on experienced cytogeneticists, although artificial intelligence-based tools can assist chromosome segmentation and metaphase image quality control to improve efficiency and reporting consistency (Chebly, 2024).

In the late 1980s, FISH marked a shift toward molecular cytogenetics by replacing radioactive probe labels with fluorescent dyes (Shakoori, 2017). FISH relies on complementary base pairing: fluorescently labeled DNA probes hybridize *in situ* to target sequences on chromosomes, and signals are visualized by fluorescence microscopy to detect SVs. With advances in probe design and labeling, FISH has expanded into multicolor formats, including multicolor FISH and spectral karyotyping. Nowadays, FISH is widely used in clinical practice for the diagnosis and evaluation of chromosomal aberrations.

Compared with karyotype analysis, FISH has a faster turnaround time because it does not require cell culture and offers higher resolution (up to 50 kb) (Cheng et al., 2025). However, due to probe design constraints and cost, clinical FISH assays are targeted rather than genome-wide and typically interrogate only regions suspected of structural variation. Therefore, FISH is often used as a second-tier diagnostic method to confirm BCRs.

### 2.2 Mate-pair sequencing

Short-read sequencing is widely used clinically, but short read length limits detection of BCR breakpoints. MPS, originally established as pairwise end sequencing in the 1990s, was developed to significantly enhance the effective length of DNA fragments (Edwards et al., 1990; Roach, 1995; Roach et al., 1995). This method involves fragmenting DNA into predetermined sizes (e.g., an insert size of 3–10 kb), circularizing the fragments, and generating reads from both ends (mates) of the DNA fragments (Korbel et al., 2007). In comparison to conventional paired-end sequencing, this technology can reach similar physical coverage with fewer sequence reads due to its larger insert sizes. While the core chemistry is well-established, recent nuances in refined circularization protocols and advanced bioinformatic algorithms have significantly enhanced its sensitivity. These modern refinements facilitate detection of breakpoint-supporting signals, which are essential for the precise detection of BCRs.

The clinical utility of this approach for identifying BCRs has been demonstrated in several studies. In a prospective prenatal study by Qian et al., MPS provides an additional 25% diagnostic yield, primarily by detecting pathogenic balanced translocations and inversions (Qian et al., 2025). This methodology provides concurrent information on both the precise genomic location and the orientation of rearrangements, which is essential for accurate clinical interpretation. The ability of MPS to resolve BCRs is further supported in reproductive medicine: Dong et al. utilized this technique to uncover cryptic BCRs linked to idiopathic male infertility, while Chau et al. successfully delineated all breakpoint junctions in azoospermia cases where karyotyping failed to provide a molecular explanation (Chau et al., 2022; Dong et al., 2023). Consequently, MPS remains a robust and cost-effective tool for comprehensive BCR assessment, though its performance can be constrained by mapping ambiguity in highly repetitive regions (Madjunkova et al., 2020).

### 2.3 Long-read sequencing

LRS generates reads of several kilobases or longer, improving detection of SVs and breakpoint resolution in complex or repetitive regions. LRS is mainly implemented through PacBio single-molecule real-time (SMRT) sequencing and Oxford Nanopore sequencing (Scarano et al., 2024). SMRT detects fluorescent signals during DNA synthesis in zero-mode waveguides (Eid et al., 2009), whereas nanopore sequencing infers bases from current changes as single-stranded DNA translocates through a nanopore (Jain et al., 2018).

In recent years, studies applying LRS in clinical research have increased rapidly. The ability to generate long reads has substantially improved the detection and breakpoint resolution of human structural variants, including BCRs, enabling broader clinical applications. For example, Chow et al. used nanopore sequencing to differentiate embryos carrying BCRs from those with a normal karyotype in 55 blastocysts from nine patients with cytogenetically confirmed reciprocal translocations. They successfully identified euploid embryos for transfer, offering a method that may be particularly useful when no informative proband is available (Chow et al., 2020). Pei et al. applied a similar strategy in preimplantation genetic testing for structural rearrangement (PGT-SR) for two patients with reciprocal translocation (Pei et al., 2021). LRS has also been used to identify other BCR types, such as inversions, in clinical research settings. Xu et al. performed LRS to investigate somatic structural variations in a cohort of 21 patients diagnosed with colorectal cancer. Notably, they discovered large-scale inversions that are often challenging to detect with short-read sequencing, which can impact the expression and structure of crucial tumor suppressor genes, including APC and CFTR (Xu et al., 2023). Together, these studies highlight the utility of LRS for resolving BCRs across diverse clinical research settings.

Despite its advantages (long reads and no PCR amplification), LRS remains more expensive than short-read sequencing, and base-calling errors can limit its clinical use (Athanasopoulou et al., 2021). With the development of circular consensus sequencing in SMRT sequencing, the accuracy of long high-fidelity reads can achieve 99.8% (Wenger et al., 2019). For ONT, R10.4 chemistry with duplex read types can generate reads with 99.9% accuracy (Kolesnikov et al., 2024). LRS has been reported to detect BCRs across a wide size range, from a few kilobases up to several megabases (Madjunkova et al., 2020; Watson et al., 2022; Xu et al., 2023).

### 2.4 Optical genome mapping

OGM is a novel cytogenetic approach for genome-wide detection of SVs, including balanced rearrangements (Lam et al., 2012). Ultra-high–molecular-weight DNA is fluorescently labeled at specific sequence motifs, linearized in nanochannels, and imaged to generate genome maps, which are then compared with a reference to detect SVs (Levy-Sakin et al., 2019). OGM has emerged as a powerful tool for identifying BCRs. Wang et al. collected samples from nine infertile balanced translocation carriers and used OGM for detection (Wang et al., 2020). The results were consistent with karyotype analysis and provided precise breakpoint intervals for the translocated chromosomes. Interestingly, they identified four new genes associated with male infertility, which may inform treatment strategies. Additionally, a recent study analyzed 154 genes from the 1000 Genomes Project using OGM, revealing 55 gene loci with complex SVs, highlighting its utility for detecting such events (Levy-Sakin et al., 2019). According to Bionano Genomics, OGM can detect balanced translocations >50 kb, inversions >30 kb, and insertions >500 bp (Levy et al., 2025).

OGM still has some limitations. First, it does not achieve base-pair resolution because it relies on long-range mapping of labeled sequence motifs rather than direct nucleotide sequencing; therefore, it cannot provide base-level sequence information or precisely resolve breakpoints and often benefits from integration with LRS or other methods for breakpoint refinement (Uppuluri et al., 2022). Second, OGM has reduced sensitivity in highly repetitive or centromeric regions, where low label density and repetitive sequence regions complicate SV detection and interpretation (Barseghyan et al., 2024). Additionally, OGM is unable to detect Robertsonian translocations. Overall, OGM provides a valuable genome-wide approach for BCR detection, but complementary technologies are often required to accurately define breakpoint locations in clinical settings.

### 2.5 Clinical decision-making workflow for BCR detection

Selecting the most appropriate technology for BCR detection by maximizing diagnostic sensitivity and precision in clinical practice requires a multi-factorial evaluation of the suspected rearrangement type, clinical urgency, and methodological characteristics. As summarized in our proposed workflow (Figure 1B), karyotyping remains the foundational first-tier test for global screening for BCRs in patients with infertility, miscarriage, or abnormal offspring due to its cost-effectiveness and genome-wide overview, a status reaffirmed by recent guidelines from ESHRE for the initial evaluation of recurrent pregnancy loss and infertility (RPL et al., 2023).

For karyotype-positive cases such as apparently balanced translocations or inversions, breakpoint confirmation and refinement may be required. In this setting, FISH provides a low-cost, hypothesis-driven approach to verify targeted regions, whereas targeted LRS can offer single-base resolution to evaluate potential gene disruptions at the junctions.

For karyotype-negative cases where a strong clinical suspicion persists (e.g., unexplained recurrent miscarriage or severe infertility), second-tier testing is necessary to identify cryptic BCRs. In this clinical scenario, MPS, OGM, and LRS provide complementary strengths. MPS is generally the most cost-effective and can localize breakpoints at near–single-base resolution, but performance may drop in highly repetitive loci and it requires more complex library preparation (Dong et al., 2023). OGM provides accurate, genome-wide detection of BCRs and complex rearrangements, but lacks single-base breakpoint resolution and is less informative in highly repetitive regions (Yin et al., 2026). LRS enables single-base breakpoint resolution and is often preferred for suspected repetitive-region breakpoints, although it is typically the highest-cost option. Accordingly, for karyotype-negative cases, MPS can serve as a cost-effective screen when repetitive-region involvement is unlikely; OGM is useful when rapid genome-wide structural overview or complex architecture is suspected; and LRS is recommended when precise breakpoint definition is required (e.g., for assessing gene disruption or positional effects or breakpoint-linked haplotyping for PGT-SR) (Xue et al., 2025).

## 3 Discussion and future perspectives

In summary, technologies for detecting BCRs have advanced substantially over the past decades. With the shift from conventional karyotype analysis in the 1960s to LRS and OGM, links between genomic features and phenotypic expression have become clearer, supporting precision medicine and personalized care. Each method has distinct strengths, limitations, and suitable clinical scenarios (Table 1). In practice, combining cytogenetic and molecular approaches remains essential to improve diagnostic accuracy, particularly for couples with unexplained infertility or recurrent miscarriage despite a normal karyotype. Overall, more comprehensive detection and finer breakpoint mapping of BCRs are increasingly feasible, and continued methodological maturation is expected to accelerate clinical translation.

**TABLE 1:** Comparison of detection techniques for BCRs.

Feature	Karyotyping	FISH	MPS	LRS	OGM
Resolution	5–10 Mb^a^	50–100 kb	1bp	1bp	500 bp–5 kb
Accuracy	Operator-dependent	>99%	>99%	>99%^b^	>99%
Turnaround time	7–14 days	24–48 h s	2 weeks	24 hrs–4 days	3–4 days
Relative cost	Low	Low-moderate	Moderate	Moderate-high	High
Major limitations	Low resolution; risk of culture failure; labor-intensive	Targeted region only; probe-dependent	Reduced performance in highly repetitive regions; complex bioinformatics analysis	High cost; platform-dependent accuracy; complex bioinformatics analysis	Cannot detect robertsonian translocations; limited sensitivity in highly repetitive regions; stringent DNA requirements

^a^ Based on 320-band level G-banding.

^b^ PacBio HiFi reads typically achieve ≥99%, and ONT R10.4 duplex reads can reach ∼99.9%.

FISH, fluorescence *in situ* hybridization; MPS, mate-pair sequencing; LRS, long read sequencing; OGM, optical genome mapping.

### 3.1 Reproductive management and genetic counseling

In assisted reproduction technology (ART), identifying BCRs is clinically important because it directly informs the design of PGT-SR and embryo transfer strategy. When the locations of BCRs are available, embryos can be classified as euploid non-carriers, euploid balanced carriers, or unbalanced. A practical transfer strategy is to prioritize euploid non-carrier embryos when available; if none are available, transfer of a euploid balanced-carrier embryo can be considered after counseling, whereas embryos predicted to be unbalanced are generally not recommended. In a retrospective study of 300 couples carrying BCRs, PGT-SR identified about 23.8% of blastocysts as both euploid and structurally balanced, and the cumulative live birth rate reached about 55.8%, supporting its utility in reducing miscarriage risk and improving outcomes (Ogur et al., 2023). Importantly, precise breakpoint identification can further enhance PGT-SR robustness by enabling haplotyping or breakpoint-spanning assays. Using proband-independent LRS in 10 *de novo* BCR families, breakpoint-phased SNP haplotyping supported embryo stratification, and prenatal diagnosis confirmed concordance in 4 cases with four healthy live births (Xue et al., 2025).

For recurrent miscarriage, identifying BCRs shifts clinical management from unexplained recurrent miscarriage to a defined chromosomal etiology, enabling structured counseling on options such as attempting natural conception with invasive prenatal diagnosis in future pregnancies, IVF with PGT-SR to avoid unbalanced conceptions, or the use of donor gametes (Motan et al., 2025). Gupta et al. showed that detecting parental balanced translocation carriers can directly guide targeted counseling and reproductive planning (Gupta et al., 2024).

### 3.2 Technical and clinical evidence gaps

Despite major progress, important limitations remain. BCRs located in highly repetitive regions, especially centromeric and pericentromeric loci, are still difficult to detect and resolve. Segmental duplications, satellite repeats, and long insertional regions are prone to fragmented assemblies and are incompletely represented in reference genomes (Espinosa et al., 2024). Short reads are often shorter than duplicated segments, resulting in ambiguous mapping and misassembly (Schluth-Bolard et al., 2025). Conventional cytogenetic methods also face inherent constraints: karyotyping has limited resolution, and FISH signals can be difficult to distinguish because of increased cross-hybridization in repetitive regions. Although LRS offers the potential to span long repetitive intervals, ultra-long read yield, coverage variability, and unresolved centromeric cores still limit consistent performance. Population-based or graph-based reference representations may help alleviate mapping ambiguity, and increasingly mature telomere-to-telomere and haplotype-resolved references are likely to provide a more robust framework for breakpoint discovery (Kolmogorov et al., 2023).

Beyond technical limitations, a major gap is the current shortage of large-scale, multicenter clinical studies and long-term follow-up data for BCR carriers. Current evidence is frequently derived from small cohorts or case series, limiting robust estimation of recurrence risks across different BCR subtypes and hampering comparative evaluation of testing strategies in real-world workflows (Qu et al., 2023; Eisfeldt et al., 2024; Liu et al., 2024). Although emerging efforts are addressing this, the field has historically lacked large-cohort validation for detecting BCRs in certain PGT-SR workflows and analytical frameworks (Zhang et al., 2025). Therefore, prospective research with standardized phenotyping and long-term outcome tracking, spanning reproductive outcomes and offspring development, remain critical to translate BCR characterization into evidence-based counseling and management (RPL et al., 2023).

### 3.3 First-tier testing debate and strategies for complex cases

In addition to these technical challenges, there is ongoing debate about how best to implement these methods in clinical workflows. The role of conventional karyotyping as first-line tools for detecting BCRs is increasingly questioned. Apparently balanced rearrangements are often associated with submicroscopic imbalances or cryptic complexity at the nucleotide level. Advocates of a genome sequencing-first strategy argue that whole-genome sequencing (WGS) using short-read sequencing can sensitively detect cryptic BCRs, refine breakpoints at base-pair resolution, and uncover unexpected complexity that is invisible to G-banding or FISH, while simultaneously detecting copy-number variants. Studies of patients with apparently balanced rearrangements and rare genetic disorders have shown that short-read WGS substantially increases diagnostic yield and could function as a first-tier test (Schluth-Bolard et al., 2019; Yu et al., 2020; Lowther et al., 2023). However, many cytogeneticists contend that karyotyping remains an irreplaceable first-line technique for BCRs because it offers an inexpensive, unbiased, genome-wide overview. Moreover, current short-read WGS pipelines continue to miss a subset of BCRs, particularly when breakpoints fall in highly repetitive or low-mappability regions (Schluth-Bolard et al., 2025), reinforcing the view that, at least for now, short-read WGS should complement rather than fully replace conventional cytogenetics in many diagnostic workflows.

To manage complex cases or uncertain results, an integrated approach may be helpful, with OGM used to provide genome-wide, long-range structural mapping and to delineate complex rearrangement patterns (Yin et al., 2026). Candidate events can be verified by MPS or LRS to refine breakpoints at nucleotide-level resolution, enabling evaluation of potential gene disruption/fusion or positional effects. For discordant findings or variants of uncertain significance, it may be advisable to seek confirmation using an independent complementary method across platforms and to incorporate family segregation analysis to clarify inheritance and interpretability (Biesecker et al., 2024). If a BCR is suspected to involve highly repetitive regions, LRS may be preferentially considered together with telomere-to-telomere (T2T) or pangenome references, because long reads can span extended repeats and directly capture junction sequences to improve detection and breakpoint resolution (Liao et al., 2023; Wang et al., 2025). In parallel, these improved reference resources provide more complete and population-representative representations of repetitive regions, reducing reference-related mapping bias and further improving breakpoint interpretation in these difficult-to-map regions.

Looking ahead, these advances are expected to shift BCR diagnostics toward routine, sequence-resolved assessment, particularly for rearrangements that have historically been challenging to interpret. Continued maturation of T2T assemblies and population-scale pangenome resources should further strengthen breakpoint interpretation by improving read alignment, reducing reference bias, and enabling more consistent characterization across diverse ancestries and genomic backgrounds. At the same time, advances in nanopore duplex sequencing and other high-accuracy, real-time long-read platforms are likely to improve analytical reliability while reducing practical barriers such as turnaround time, throughput constraints, and informatics complexity, making comprehensive, base-pair-level characterization of BCRs increasingly feasible in routine clinical laboratories. Together, these innovations should enable truly genome-wide, sequence-resolved diagnostics for individuals carrying BCRs, facilitating clearer genotype-phenotype correlations and bridging the gap between research applications and clinical implementation.

## References

[B1] Amor D. J. Gardner R. M. (2025). Gardner and sutherland's chromosome abnormalities and genetic counseling. Oxford University Press.

[B2] Arsham M. S. Margaret J. Lawce H. J. (2017). The AGT cytogenetics laboratory manual. Canada: Blackwell.

[B3] Athanasopoulou K. Boti M. A. Adamopoulos P. G. Skourou P. C. Scorilas A. (2021). Third-generation sequencing: the spearhead towards the radical transformation of modern genomics. Life (Basel) 12, 30. 10.3390/life12010030 35054423 PMC8780579

[B4] Barseghyan H. Eisenreich D. Lindt E. Wendlandt M. Scharf F. Benet-Pages A. (2024). Optical genome mapping as a potential routine clinical diagnostic method. Genes (Basel) 15, 398. 10.3390/genes15030342 38540401 PMC10970541

[B5] Biesecker L. G. Byrne A. B. Harrison S. M. Pesaran T. Schäffer A. A. Shirts B. H. (2024). ClinGen guidance for use of the PP1/BS4 co-segregation and PP4 phenotype specificity criteria for sequence variant pathogenicity classification. Am. J. Hum. Genet. 111, 24–38. 10.1016/j.ajhg.2023.11.009 38103548 PMC10806742

[B6] Chau M. H. K. Li Y. Dai P. Shi M. Zhu X. Wah Chung J. P. (2022). Investigation of the genetic etiology in Male infertility with apparently balanced chromosomal structural rearrangements by genome sequencing. Asian J. Androl. 24, 248–254. 10.4103/aja2021106 35017386 PMC9226698

[B7] Chebly A. (2024). Cancer cytogenetics in the era of artificial intelligence: shaping the future of chromosome analysis. Future Oncol. 20, 2303–2305. 10.1080/14796694.2024.2385296 39129712 PMC11520557

[B8] Cheng L. Davidson D. D. Zhang S. (2025). Genomic aberration detection by fluorescence *in situ* hybridization. Hum. Pathol. 165, 105906. 10.1016/j.humpath.2025.105906 40782985

[B9] Chow J. F. C. Cheng H. H. Y. Lau E. Y. L. Yeung W. S. B. Ng E. H. Y. (2020). Distinguishing between carrier and noncarrier embryos with the use of long-read sequencing in preimplantation genetic testing for reciprocal translocations. Genomics 112, 494–500. 10.1016/j.ygeno.2019.04.001 30946890

[B10] Dong Z. Yan J. Xu F. Yuan J. Jiang H. Wang H. (2019). Genome sequencing explores complexity of chromosomal abnormalities in recurrent miscarriage. Am. J. Hum. Genet. 105, 1102–1111. 10.1016/j.ajhg.2019.10.003 31679651 PMC6904795

[B11] Dong Z. Qian J. Law T. S. M. Chau M. H. K. Cao Y. Xue S. (2023). Mate-pair genome sequencing reveals structural variants for idiopathic Male infertility. Hum. Genet. 142, 363–377. 10.1007/s00439-022-02510-4 36526900

[B12] Edwards A. Voss H. Rice P. Civitello A. Stegemann J. Schwager C. (1990). Automated DNA sequencing of the human HPRT locus. Genomics 6, 593–608. 10.1016/0888-7543(90)90493-e 2341149

[B13] Eid J. Fehr A. Gray J. Luong K. Lyle J. Otto G. (2009). Real-time DNA sequencing from single polymerase molecules. Science 323, 133–138. 10.1126/science.1162986 19023044

[B14] Eisfeldt J. Ameur A. Lenner F. Ten Berk De Boer E. Ek M. Wincent J. (2024). A national long-read sequencing study on chromosomal rearrangements uncovers hidden complexities. Genome Res. 34, 1774–1784. 10.1101/gr.279510.124 39472022 PMC11610602

[B15] Espinosa E. Bautista R. Larrosa R. Plata O. (2024). Advancements in long-read genome sequencing technologies and algorithms. Genomics 116, 110842. 10.1016/j.ygeno.2024.110842 38608738

[B16] Gupta A. Dhanvij M. Gangane N. Bansal P. Davile M. Shende U. (2024). Prenatal diagnosis of unbalanced translocations in recurrent pregnancy losses in two couples. J. Fetal Med. 11, 233–236. 10.1055/s-0045-1808068

[B17] Jain M. Koren S. Miga K. H. Quick J. Rand A. C. Sasani T. A. (2018). Nanopore sequencing and assembly of a human genome with ultra-long reads. Nat. Biotechnol. 36, 338–345. 10.1038/nbt.4060 29431738 PMC5889714

[B18] Karger (2024). ISCN 2024 - An International System for Human Cytogenomic Nomenclature (2024). 10.1159/000538512 39571546

[B19] Kolesnikov A. Cook D. Nattestad M. Brambrink L. Mcnulty B. Gorzynski J. (2024). Local read haplotagging enables accurate long-read small variant calling. Nat. Commun. 15, 5907. 10.1038/s41467-024-50079-5 39003259 PMC11246426

[B20] Kolmogorov M. Billingsley K. J. Mastoras M. Meredith M. Monlong J. Lorig-Roach R. (2023). Scalable nanopore sequencing of human genomes provides a comprehensive view of haplotype-resolved variation and methylation. Nat. Methods 20, 1483–1492. 10.1038/s41592-023-01993-x 37710018 PMC11222905

[B21] Korbel J. O. Urban A. E. Affourtit J. P. Godwin B. Grubert F. Simons J. F. (2007). Paired-end mapping reveals extensive structural variation in the human genome. Science. 318 **,** 420–426. 10.1126/science.1149504 17901297 PMC2674581

[B22] Lam E. T. Hastie A. Lin C. Ehrlich D. Das S. K. Austin M. D. (2012). Genome mapping on nanochannel arrays for structural variation analysis and sequence assembly. Nat. Biotechnol. 30, 771–776. 10.1038/nbt.2303 22797562 PMC3817024

[B23] Levy B. Wapner R. (2018). Prenatal diagnosis by chromosomal microarray analysis. Fertil. Steril. 109, 201–212. 10.1016/j.fertnstert.2018.01.005 29447663 PMC5856154

[B24] Levy B. Burnside R. D. Akkari Y. (2025). Optical genome mapping: a new tool for cytogenomic analysis. Genes 16, 924. 10.3390/genes16080924 40869972 PMC12385997

[B25] Levy-Sakin M. Pastor S. Mostovoy Y. Li L. Leung A. K. Y. Mccaffrey J. (2019). Genome maps across 26 human populations reveal population-specific patterns of structural variation. Nat. Commun. 10, 1025. 10.1038/s41467-019-08992-7 30833565 PMC6399254

[B26] Liao W.-W. Asri M. Ebler J. Doerr D. Haukness M. Hickey G. (2023). A draft human pangenome reference. Nature 617, 312–324. 10.1038/s41586-023-05896-x 37165242 PMC10172123

[B27] Liu D. Chen C. Huang Q. Dong Y. Xu L. Dong M. (2024). Preimplantation genetic testing for complex chromosomal rearrangements: clinical outcomes and potential risk factors. Front. Genet. 15, 1401549. 10.3389/fgene.2024.1401549 39139821 PMC11320417

[B28] Lowther C. Valkanas E. Giordano J. L. Wang H. Z. Currall B. B. O’keefe K. (2023). Systematic evaluation of genome sequencing for the diagnostic assessment of autism spectrum disorder and fetal structural anomalies. Am. J. Hum. Genet. 110, 1454–1469. 10.1016/j.ajhg.2023.07.010 37595579 PMC10502737

[B29] Madjunkova S. Sundaravadanam Y. Antes R. Abramov R. Chen S. Yin Y. (2020). Detection of structural rearrangements in embryos. N. Engl. J. Med. 382, 2472–2474. 10.1056/NEJMc1913370 32558475

[B30] Motan T. Cockwell H. Elliott J. Antaki R. White J. (2025). Guideline no. 464: recurrent pregnancy loss. J. Obstet. Gynaecol. Can. 47, 103167. 10.1016/j.jogc.2025.103167 41176277

[B31] Ogur C. Kahraman S. Griffin D. K. Cinar Yapan C. Tufekci M. A. Cetinkaya M. (2023). PGT for structural chromosomal rearrangements in 300 couples reveals specific risk factors but an interchromosomal effect is unlikely. Reprod. Biomed. Online 46, 713–727. 10.1016/j.rbmo.2022.07.016 36803887

[B32] Pei Z. Deng K. Lei C. Du D. Yu G. Sun X. (2021). Identifying balanced chromosomal translocations in human embryos by Oxford nanopore sequencing and breakpoints region analysis. Front. Genet. 12, 810900. 10.3389/fgene.2021.810900 35116057 PMC8804325

[B33] Poot M. Hochstenbach R. (2021). Prevalence and phenotypic impact of robertsonian translocations. Mol. Syndromol. 12, 1–11. 10.1159/000512676 33776621 PMC7983559

[B34] Qian J. Wang H. Liang H. Zheng Y. Yu M. Tse W. T. (2025). Mate-pair sequencing enables identification and delineation of balanced and unbalanced structural variants in prenatal cytogenomic diagnostics. Clin. Chem. 71, 155–168. 10.1093/clinchem/hvae146 39749521

[B35] Qu J. Li S. Yu D. (2023). Detection of complex chromosome rearrangements using optical genome mapping. Gene 884, 147688. 10.1016/j.gene.2023.147688 37543218

[B36] Roach J. C. (1995). Random subcloning. Genome Res. 5, 464–473. 10.1101/gr.5.5.464 8808467

[B37] Roach J. C. Boysen C. Wang K. Hood L. (1995). Pairwise end sequencing: a unified approach to genomic mapping and sequencing. Genomics 26, 345–353. 10.1016/0888-7543(95)80219-c 7601461

[B38] Rpl T. E. G. G. O. Bender Atik R. Christiansen O. B. Elson J. Kolte A. M. Lewis S. (2023). ESHRE guideline: recurrent pregnancy loss: an update in 2022. Hum. Reprod. Open. 2023, hoad002. 10.1093/hropen/hoad002 36873081 PMC9982362

[B39] Saldarriaga W. García-Perdomo H. A. Arango-Pineda J. Fonseca J. (2015). Karyotype *versus* genomic hybridization for the prenatal diagnosis of chromosomal abnormalities: a metaanalysis. Am. J. Obstetrics And Gynecol. 212, 330.e331. 10.1016/j.ajog.2014.10.011 25305409

[B40] Satkin N. B. Karaman B. Ergin S. Kayserili H. Kalelioglu I. H. Has R. (2020). Array-comparative genomic hybridization results in clinically affected cases with apparently balanced chromosomal rearrangements. Balk. J. Med. Genet. 23, 25–34. 10.2478/bjmg-2020-0026 33816069 PMC8009573

[B41] Scarano C. Veneruso I. De Simone R. R. Di Bonito G. Secondino A. D'argenio V. (2024). The third-generation sequencing challenge: novel insights for the omic sciences. Biomolecules 14, 568. 10.3390/biom14050568 38785975 PMC11117673

[B42] Schluth-Bolard C. Diguet F. Chatron N. Rollat-Farnier P. A. Bardel C. Afenjar A. (2019). Whole genome paired-end sequencing elucidates functional and phenotypic consequences of balanced chromosomal rearrangement in patients with developmental disorders. J. Med. Genet. 56, 526–535. 10.1136/jmedgenet-2018-105778 30923172

[B43] Schluth-Bolard C. El Khattabi L. Rollat-Farnier P. A. Chatron N. Beaumont M. Reynaud N. (2025). Resolving structural variations missed by short-read sequencing uncovers their pathogenicity. J. Med. Genet. 62, 750–757. 10.1136/jmg-2025-110838 40835298

[B44] Scriven P. N. Handyside A. H. Ogilvie C. M. (1998). Chromosome translocations: segregation modes and strategies for preimplantation genetic diagnosis. Prenat. Diagn 18, 1437–1449. 10.1002/(SICI)1097-0223(199812)18:13<1437::AID-PD497>3.0.CO;2-P 9949444

[B45] Shakoori A. R. (2017). Fluorescence *in situ* hybridization (FISH) and its applications. Chromosome Struct. Aberrations 10, 343–367. 10.1007/978-81-322-3673-3_16

[B46] Shen J. Ding T. Sun X. Yang J. Zhang Y. Wang J. (2024). Comprehensive analysis of genomic complexity in the 5' end coding region of the DMD gene in patients of exons 1-2 duplications based on long-read sequencing. BMC Genomics 25, 292. 10.1186/s12864-024-10224-2 38504154 PMC10949565

[B47] Uppuluri L. Wang Y. Young E. Wong J. S. Abid H. Z. Xiao M. (2022). Multiplex structural variant detection by whole-genome mapping and nanopore sequencing. Sci. Rep. 12, 6512. 10.1038/s41598-022-10483-7 35444207 PMC9021263

[B48] Wang H. Jia Z. Mao A. Xu B. Wang S. Wang L. (2020). Analysis of balanced reciprocal translocations in patients with subfertility using single-molecule optical mapping. J. Assisted Reproduction Genet. 37, 509–516. 10.1007/s10815-020-01702-z 32026199 PMC7125258

[B49] Wang B. Jia P. Bush S. J. Wang X. Yang Y. Zhang Y. (2025). A telomere-to-telomere diploid reference genome and centromere structure of the Chinese quartet. Genomics, Proteomics & Bioinforma., qzaf118. 10.1093/gpbjnl/qzaf118 41298323 PMC13075991

[B50] Watson C. M. Holliday D. L. Crinnion L. A. Bonthron D. T. (2022). Long-read nanopore DNA sequencing can resolve complex intragenic duplication/deletion variants, providing information to enable preimplantation genetic diagnosis. Prenat. Diagn 42, 226–232. 10.1002/pd.6089 35014072 PMC9305782

[B51] Wenger A. M. Peluso P. Rowell W. J. Chang P.-C. Hall R. J. Concepcion G. T. (2019). Accurate circular consensus long-read sequencing improves variant detection and assembly of a human genome. Nat. Biotechnol. 37, 1155–1162. 10.1038/s41587-019-0217-9 31406327 PMC6776680

[B52] Xia Q. Li S. Ding T. Liu Z. Liu J. Li Y. (2023). Nanopore sequencing for detecting reciprocal translocation carrier status in preimplantation genetic testing. BMC Genomics 24, 1. 10.1186/s12864-022-09103-5 36593441 PMC9809107

[B53] Xu L. Wang X. Lu X. Liang F. Liu Z. Zhang H. (2023). Long-read sequencing identifies novel structural variations in colorectal cancer. PLoS Genet. 19, e1010514. 10.1371/journal.pgen.1010514 36812239 PMC10013895

[B54] Xue J. Xie M. Cai J. Kang K. Gu M. Li M. (2025). ViLR: a novel virtual long read method for breakpoint identification and direct SNP haplotyping in *de novo* PGT-SR carriers without a proband. Reproductive Biol. Endocrinol. 23, 34. 10.1186/s12958-025-01366-3 40038676 PMC11881346

[B55] Yin K. Lü Y. Zhang H. Li M. Chang J. Yang X. (2026). Evaluation of the efficacy of optical genome mapping in prenatal diagnosis: a retrospective cohort study. J. Transl. Med. 24, 89. 10.1186/s12967-025-06901-9 41559681 PMC12822095

[B56] Yu M. H. C. Chau J. F. T. Au S. L. K. Lo H. M. Yeung K. S. Fung J. L. F. (2020). Evaluating the clinical utility of genome sequencing for cytogenetically balanced chromosomal abnormalities in prenatal diagnosis. Front. Genet. 11, 620162. 10.3389/fgene.2020.620162 33584815 PMC7873444

[B57] Zhang S. Liang F. Lei C. Wu J. Fu J. Yang Q. (2019). Long-read sequencing and haplotype linkage analysis enabled preimplantation genetic testing for patients carrying pathogenic inversions. J. Med. Genet. 56, 741–749. 10.1136/jmedgenet-2018-105976 31439719 PMC6860410

[B58] Zhang S. Lei C. Wu J. Zhou J. Xiao M. Zhu S. (2021). Meiotic heterogeneity of trivalent structure and interchromosomal effect in blastocysts with robertsonian translocations, Front. Genet. 12. 609563. 10.3389/fgene.2021.609563 33679881 PMC7928295

[B59] Zhang S. Pei Z. Lei C. Zhu S. Deng K. Zhou J. (2023). Detection of cryptic balanced chromosomal rearrangements using high-resolution optical genome mapping. J. Med. Genet. 60, 274–284. 10.1136/jmedgenet-2022-108553 35710108

[B60] Zhang S. Gao Y. Wang X. Li Q. Tan J. Liang B. (2025). Preimplantation genetic testing for structural rearrangements by genome-wide SNP genotyping and haplotype analysis: a prospective multicenter clinical study. eBioMedicine 111, 105514. 10.1016/j.ebiom.2024.105514 39708428 PMC11731775

[B61] Zhu S. Zhu Y. Zhang F. Wu J. Chen Y. Sun Y. (2022). FISH analysis of numerical chromosomal abnormalities in the sperm of robertsonian translocation der(13;14)(q10;q10) carriers. Front. Genet. 13, 1010568. 10.3389/fgene.2022.1010568 36238152 PMC9551382

